# Transcriptional response profiles of paired tumor-normal samples offer novel perspectives in pan-cancer analysis

**DOI:** 10.18632/oncotarget.17295

**Published:** 2017-04-20

**Authors:** Shuofeng Hu, Hanyu Yuan, Zongcheng Li, Jian Zhang, Jiaqi Wu, Yaowen Chen, Qiang Shi, Wu Ren, Ningsheng Shao, Xiaomin Ying

**Affiliations:** ^1^ Beijing Institute of Basic Medical Sciences, Beijing 100850, China; ^2^ Translational Medicine Center of Stem Cells, 307-Ivy Translational Medicine Center, Laboratory of Oncology, Affiliated Hospital, Academy of Military Medical Sciences, Beijing 100071, China; ^3^ Department of Obstetrics and Gynecology, Fuzhou General Hospital of Nanjing Military Command, Fujian 350025, China; ^4^ Department of Gastrointestinal Surgery, The First Affiliated Hospital of Jilin University, Changchun 130021, China

**Keywords:** transcriptional response profiles, paired tumor-normal sample, pan-cancer, comparison, biomarker

## Abstract

Both tumor and adjacent normal tissues are valuable in cancer research. Transcriptional response profiles represent the changes of gene expression levels between paired tumor and adjacent normal tissues. In this study, we performed a pan-cancer analysis based on the transcriptional response profiles from 633 samples across 13 cancer types. We obtained two interesting results. Using consensus clustering method, we characterized ten clusters with distinct transcriptional response patterns and enriched pathways. Notably, head and neck squamous cell carcinoma was divided in two subtypes, enriched in cell cycle-related pathways and cell adhesion-related pathways respectively. The other interesting result is that we identified 92 potential pan-cancer genes that were consistently upregulated across multiple cancer types. Knockdown of FAM64A or TROAP inhibited the growth of cancer cells, suggesting that these genes may promote tumor development and are worthy of further validations. Our results suggest that transcriptional response profiles of paired tumor-normal tissues can provide novel perspectives in pan-cancer analysis.

## INTRODUCTION

Cancer accounted for approximately 8.2 million deaths in 2012. About 14.1 million new cancer cases occur globally each year [1]. Cancer is typically a genetic disease derived from genome aberrances such as somatic mutations, copy-number alterations, DNA methylations, and gene fusions [2]. In recent years, there are growing evidences that genomic molecular characteristics can classify patients with distinct clinical outcomes and contribute to the development of precision medicine [3–9]. For example, PAM50, a widely used breast cancer classifier based on gene expression profile, can divide patients into five subtypes corresponding to different clinical outcomes [3]. By examining the expression levels of specific target molecules (e.g. HER2), targeted therapy such as trastuzumab and pertuzumab can inhibit tumor growth by interfering with these cancer driver genes [4, 5]. The Cancer Genome Atlas (TCGA) Research Network [10] has reported a series of genome-wide studies in which cancer heterogeneity within single cancer type is well described at the molecular level and most cancer types possess multiple subtypes with distinct molecular characteristics [6, 7, 9].

On the other hand, there are also common alterations of cancer-related genes (e.g. EGFR) and pathways (e.g. the p53 pathway) [11] which are shared across different cancer types or subtypes. These facts have led to the “pan-cancer” analysis which integrates various cancer types [11–14]. For example, by integrating thousands of genetic and epigenetic features, 3,299 TCGA tumors from 12 cancer types were classified into two major classes which were dominated by mutations and copy number changes, respectively [15]. Furthermore, cancer therapies may also benefit from pan-cancer analysis by targeting the driving molecular events despite tissue origin [16–18]. For example, a fraction of non-small cell lung carcinoma (NSCLC), inflammatory myofibroblastic tumor, and anaplastic large cell lymphoma, which share ALK fusions, can be treated with ALK inhibitors and this strategy has shown clinical efficacy [16].

As is known, normal tissues adjacent to tumor is valuable for cancer research. Studies on mutations, structural variations, or DNA copy number alterations have demonstrated the value of normal tissues in identifying cancer-associated genome variations accurately [19]. Accumulative evidences have demonstrated that transcriptome from adjacent normal tissue is also valuable in cancer classification and biomarker discovery [20, 21]. One example is that a reproducible gene expression signature correlated with survival in patients with hepatocellular carcinoma (HCC) was derived from tumor-adjacent normal tissues, while tumor tissues failed to yield significant results [21].

Nonetheless, the impact of involving normal tissues in transcriptome-based pan-cancer analysis is still unknown. In this study, we performed a systematic analysis based on transcriptional response profiles of 633 paired tumor-normal tissue samples from 13 cancer types available in TCGA. Transcriptional response profiles of paired tumor and adjacent normal tissues can reduce both individual differences and the impact of tissue-specific genes. Our analysis identified some interesting results that were different from tumor-only pan-cancer studies. Ten clusters with distinct molecular features were distinguished and one of them contained four cancer types. Head and neck squamous (HNSC) samples were divided into two subtypes, one enriched in cell cycle-related pathways and the other enriched in cell adhesion-related pathways. Furthermore, 92 genes were consistently upregulated in multiple cancer types compared to adjacent normal tissues. Knockdown of two of these genes, FAM64A and TROAP, in MDA-MB-231 cell line inhibited cancer cell growth. Our results suggest that involvement of paired tumor-normal tissues may provide novel perspectives in pan-cancer analysis.

## RESULTS

### Transcriptional response profile-based pan-cancer clustering

We collected gene expression profiles of 633 paired tumor-normal samples from 13 TCGA cancer data sets [6–9, 22–29]: bladder urothelial carcinoma (BLCA, n = 19), breast cancer (BRCA, n = 111), colon adenocarcinoma (COAD, n = 41), head and neck squamous cell carcinoma (HNSC, n = 41), kidney chromophobe renal cell carcinoma (KICH, n = 25), kidney clear cell renal cell carcinoma (KIRC, n = 72), kidney renal papillary cell carcinoma (KIRP, n = 32), liver hepatocellular carcinoma (LIHC, n = 50), lung adenocarcinoma (LUAD, n = 57), lung squamous cell carcinoma (LUSC, n = 51), prostate adenocarcinoma (PRAD, n = 52), thyroid carcinoma (THCA, n = 59), and uterine corpus endometrial carcinoma (UCEC, n = 23) (Supplementary Table 1). The gene expression profiles from tumor and normal tissues were processed according to the previous pan-cancer analysis [11, 13]. Transcriptional responses were represented by the log2(fold-change) of gene expression levels from paired tumor and normal samples. Genes with log2(fold-change) ≥ 2 in at least 10% of all samples were retained for subsequent analysis. Clustering results derived from other proximal cutoff values were consistent (Supplementary Figure 1).

Then we applied consensus clustering algorithm [30] to characterize transcriptional response profiles in paired tissue-normal analysis. We performed consensus clustering on the 633 cancer samples with clustering number (*i*.*e*. *k*) varying from 2 to 20 to determine the optimal *k* (see Methods). Considering the result of the Δ(*k*) *vs k* plot (Supplementary Figure 2A) and the heatmap (Supplementary Figure 2B), *k* = 10 was determined as the final cluster number.

**Figure 1:**
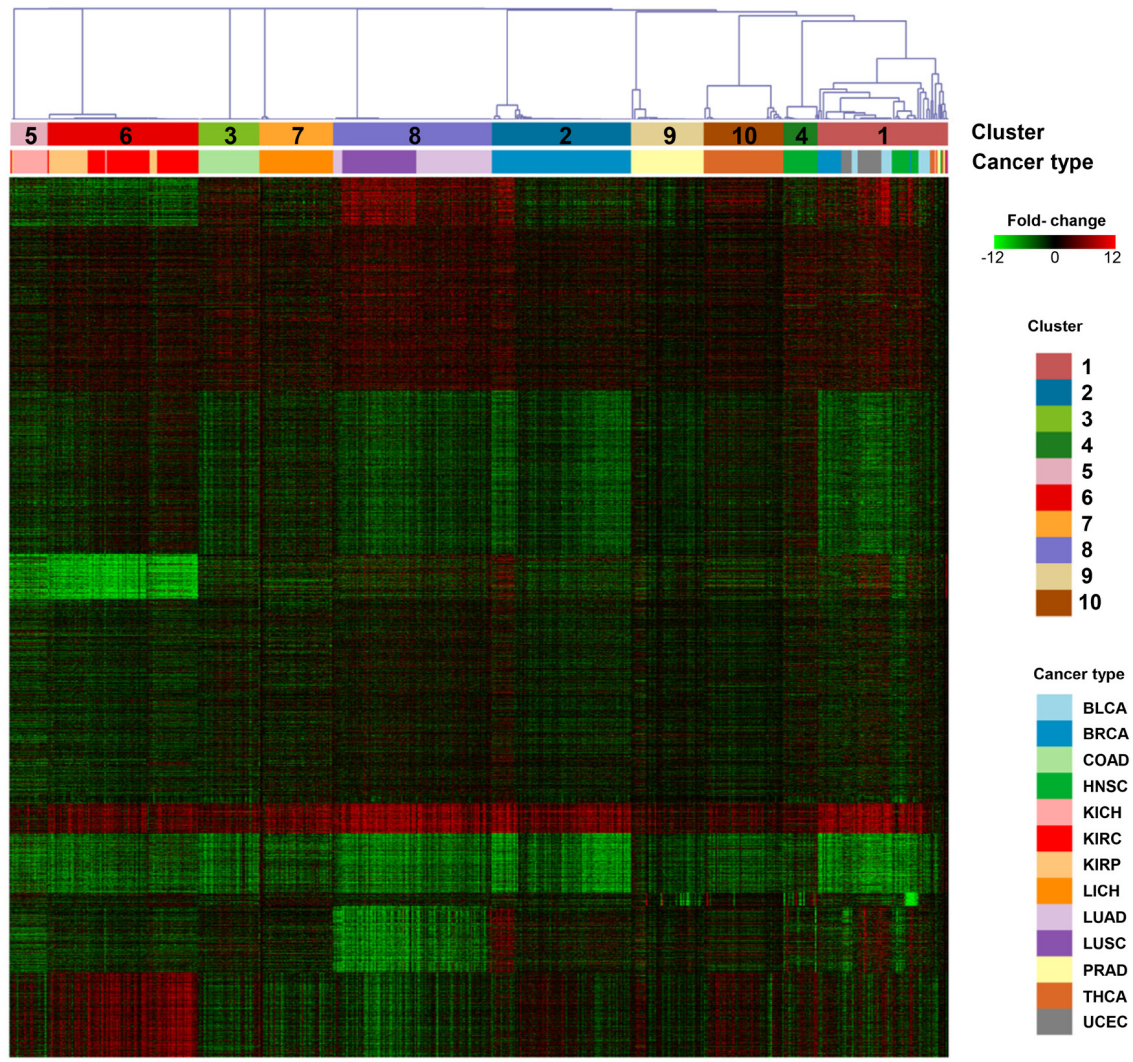
Consensus clustering result of 633 paired tumor-normal samples Heatmap shows the pattern of transcriptional response profiles derived from consensus clustering algorithm. Rows indicate genes and columns indicate 633 samples from 13 cancer types. Red color indicates positive transcriptional responses while green color indicates negative transcriptional responses. The 10 clusters identified are shown by different colors in the top bar with 1 to 10 marked on it. Cancer types are shown by different colors in the second bar.

Consensus clustering result at *k* = 10 was illustrated by the dendrogram and the heatmap of transcriptional response profiles (Figure 1, Table 1). The first cluster C1, mainly involved four cancer types: BLCA (19/19), BRCA (17/111), HNSC (18/41) and UCEC (23/23). Seven clusters were dominated by single cancer types: C2 BRCA, C3 COAD, C4 HNSC, C5 KICH, C7 LIHC, C9 PRAD and C10 THCA. The last two clusters C6 and C8 both contained two cancer types originating from the same organ. C6 was composed of KIRC samples (70/72) and KIRP samples (31/32). C8 was composed of LUAD samples (57/57) and LUSC samples (50/51). Samples of BRCA and HNSC were both split into two clusters. Interestingly, the BRCA samples classified into C1 were all basal-like breast cancers (17/17), whereas the rest BRCA samples clustered in C2 were luminal and HER2-positive subtypes. On the other hand, KICH, a cancer type derived from renal tissue, did not cluster with C6, which was composed of KIRC and KIRP.

**Table 1:** The 13 cancer types and their relationship to 10 clusters derived from transcriptional response-based method

Handle	C1	C2	C3	C4	C5	C6	C7	C8	C9	C10	Total
**BLCA**	19	0	0	0	0	0	0	0	0	0	**19**
**BRCA**	17	94	0	0	0	0	0	0	0	0	**111**
**COAD**	0	0	41	0	0	0	0	0	0	0	**41**
**HNSC**	18	0	0	23	0	0	0	0	0	0	**41**
**KICH**	1	0	0	0	23	1	0	0	0	0	**25**
**KIRC**	1	0	0	0	1	70	0	0	0	0	**72**
**KIRP**	0	0	0	0	1	31	0	0	0	0	**32**
**LIHC**	0	0	0	0	0	0	50	0	0	0	**50**
**LUAD**	0	0	0	0	0	0	0	57	0	0	**57**
**LUSC**	1	0	0	0	0	0	0	50	0	0	**51**
**PRAD**	3	0	0	0	0	0	0	0	49	0	**52**
**THCA**	5	0	0	0	0	0	0	0	0	54	**59**
**UCEC**	23	0	0	0	0	0	0	0	0	0	**23**
**Total**	**88**	**94**	**41**	**23**	**25**	**102**	**50**	**107**	**49**	**54**	**633**

We also performed consensus clustering algorithm on expression profiles from tumor samples (Supplementary Figure 3A). The result was consistent with that in previous pan-cancer reports derived from tumor-only samples [11, 13]. There were three significant differences in the clustering pattern comparing with that derived from paired samples. First, HNSC resembled BLCA and LUSC and they formed a cluster in tumor-only clustering. Second, one cluster consisted of BLCA (18/19), HNSC (41/41) and LUSC (41/51) in tumor-only clustering. Third, LUAD and LUSC were separated in tumor-only clustering. The clustering differences between tumor-only and paired pan-cancer analysis suggest that individual differences and tissue specificity have some effects on pan-cancer analysis results, which should be investigated further.

### Differentially regulated genes and pathway analysis across 10 clusters

**Figure 2:**
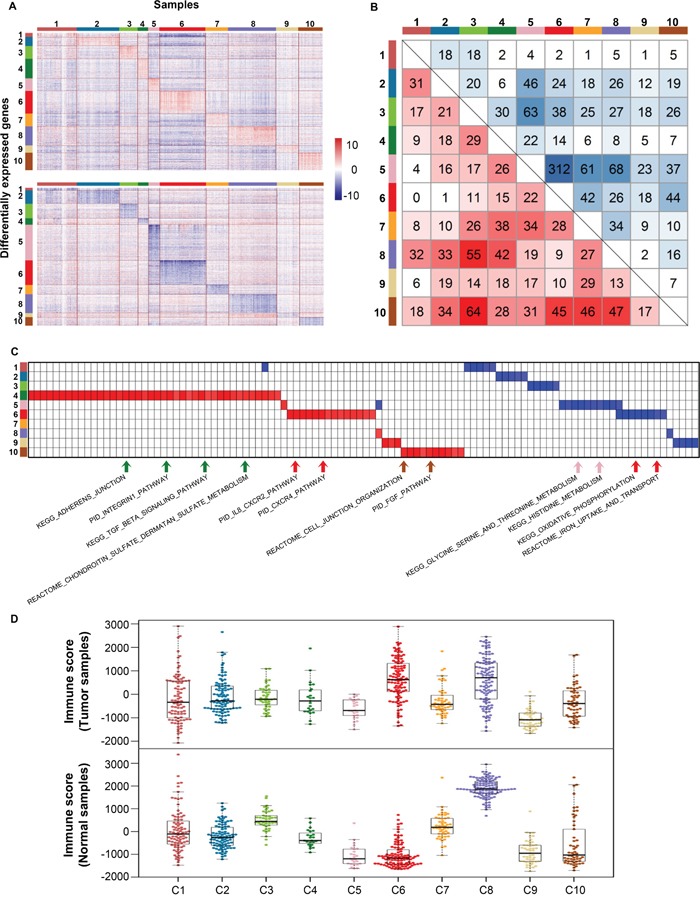
Differentially expressed genes and pathway analysis across 10 clusters **(A)** Heatmaps show upregulated and downregulated genes of each cluster on the top and bottom, respectively. Rows indicate genes differentially expressed in corresponding clusters and columns indicate samples sorted by clusters. **(B)** Overlapping genes between each two clusters. Red color means overlaps derived from upregulated genes while blue color means overlaps derived from downregulated genes. **(C)** GSEA heatmap shows pathways with nominal *p* value < 0.01. Red color indicates upregulated pathways and blue color indicates downregulated pathways. Representative pathways were pointed out below. **(D)** Immune scores across 10 clusters in both tumor and normal samples.

To investigate the transcriptional response differences among the 10 clusters, we compared the transcriptional response profiles of each cluster with the other clusters. Genes with *p* value < 0.01 and log2(fold-change) ≥ 2 were selected as upregulated genes while genes with *p* value < 0.01 and log2(fold-change) ≤ -2 were selected as highly downregulated genes (Figure 2A). The numbers of upregulated genes of each cluster ranged from 135 (C1) to 767 (C6). Meanwhile, the numbers of downregulated genes of each cluster ranged from 98 (C1) to 1343 (C5). Next, we analyzed the overlapping genes between each two clusters for upregulated and downregulated genes (Figure 2B). C5 and C6 exhibited the largest number of overlapping downregulated genes (312) and both of them originated from kidney tissue. However, the other clusters showed only small numbers of overlapping upregulated or downregulated genes.

We next identified pathways specifically enriched in each cluster using gene set enrichment analysis (GSEA) method [31] (Figure 2C). C4 contained the largest number of upregulated pathways, while C5 contained the largest number of downregulated pathways. Particularly, we observed an obvious enrichment of upregulated pathways related to cell adhesion and motion in C4-HNSC. Cell adhesion and traction can promote cell migration process which allows tumor metastasis through the circulatory system. Survival analysis revealed that C4 had the worst prognosis in 10 clusters (Supplementary Figure 4). We also found that ‘immune system’-related pathways were upregulated in C6-KIRC/KIRP, which suggests that immune system is activated in this cluster (Figure 2C). It is possible that patients in C6-KIRC/KIRP may respond to immunotherapeutics such as anti-PD1/PDL1 and anti-CTLA4 which are promising strategies in treatment of advanced melanoma and other tumor types [32–35]. While in another renal carcinoma-related cluster, C5-KICH, amino acid metabolism-related pathways were downregulated, which may prevent tumor cell from rapid cellular proliferation [36].

We further evaluated the levels of immune cells in both tumor and normal samples using ESTIMATE method [37] (Figure 2D). Among tumor samples, immune scores were significantly higher in both C6-KIRC/KIRP and C8-LUAD/LUSC than those in the other clusters (C6 posthoc Maximum *p* value = 3.3E-06, C8 posthoc Maximum *p* value = 2.1E-05). However, among normal samples, C6 exhibited a low immune score while C8 had the highest immune score. Since lung is an organ exchanging gas with the outside world, it is more vulnerable to foreign particles and microbes. This may result in the high immune scores of both tumor and normal tissues in C8.

### Subtyping of head and neck squamous cell carcinoma

**Figure 3:**
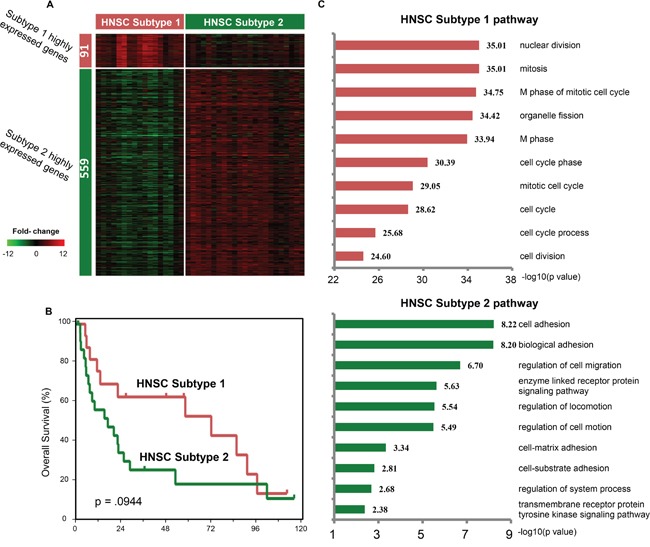
Comparison of two HNSC subtypes **(A)** The heatmap shows transcriptional response profiles of differentially expressed genes between two HNSC subtypes. Rows indicate genes and columns indicate HNSC samples. The numbers of differentially expressed genes are labeled on the left bar. **(B)** Kaplan-Meier analysis for overall survival between two HNSC subtypes. **(C)** DAVID gene functional annotation of differentially expressed genes in each HNSC subtypes. GO BP terms are ranked according to their negative log10-transformed *p* values. Top 10 GO biological process terms are shown in bar plots.

In the clustering result, HNSC samples were split into two clusters (Figure 2A), which was not observed in tumor-only analysis (Supplementary Figure 3A). One isolated HNSC sample far from the other HNSCs in C1 was excluded from further analysis (Supplementary Figure 5). We compared the transcriptional response profiles from the two subtypes, which referred to HNSC Subtype 1 (17 samples in C1) and HNSC Subtype 2 (23 samples in C4). We identified 91 and 559 upregulated genes in the two HNSC subtypes, respectively (Figure 3A). We then investigated the relevance of these subtypes with clinical prognosis. The survival curves clearly separated them from the beginning to 8 years. The median survival time of Subtype 1 and Subtype 2 were 72.2 months and 15.98 months, respectively, although *p* value is not significant (*p* value = 0.0944, Figure 3B). We hypothesized that the non-significant survival difference might be ascribed to the small number of HNSC samples.

To uncover the underlying mechanisms, we used DAVID Gene Functional Annotation Tool [38, 39] on the upregulated genes and found different enriched pathways between the two subtypes (Figure 3C). HNSC Subtype 1 was highly enriched in cell cycle-related pathways that control tumor proliferation, while HNSC Subtype 2 was highly enriched in cell adhesion- and motility-related pathways, which may lead to metastasis and invasion. Since tumors that overexpress cell cycle genes are sensitive to chemotherapy, HNSC subtype 1 may be suitable for chemotherapy.

Next, gene mutations in HNSC Subtype 1 and Subtype 2 were examined. We found that apoptosis and cell cycle regulator TP53 harbored a high number of mutations in both two subtypes (Supplementary Figure 6A). However, no gene mutation presented a significant level of enrichment in either subtypes (Supplementary Figure 6B). This phenomenon indicates that differences between HNSC Subtype 1 and Subtype 2 may not result from gene mutations.

### Upregulated genes across multiple cancer types

**Figure 4:**
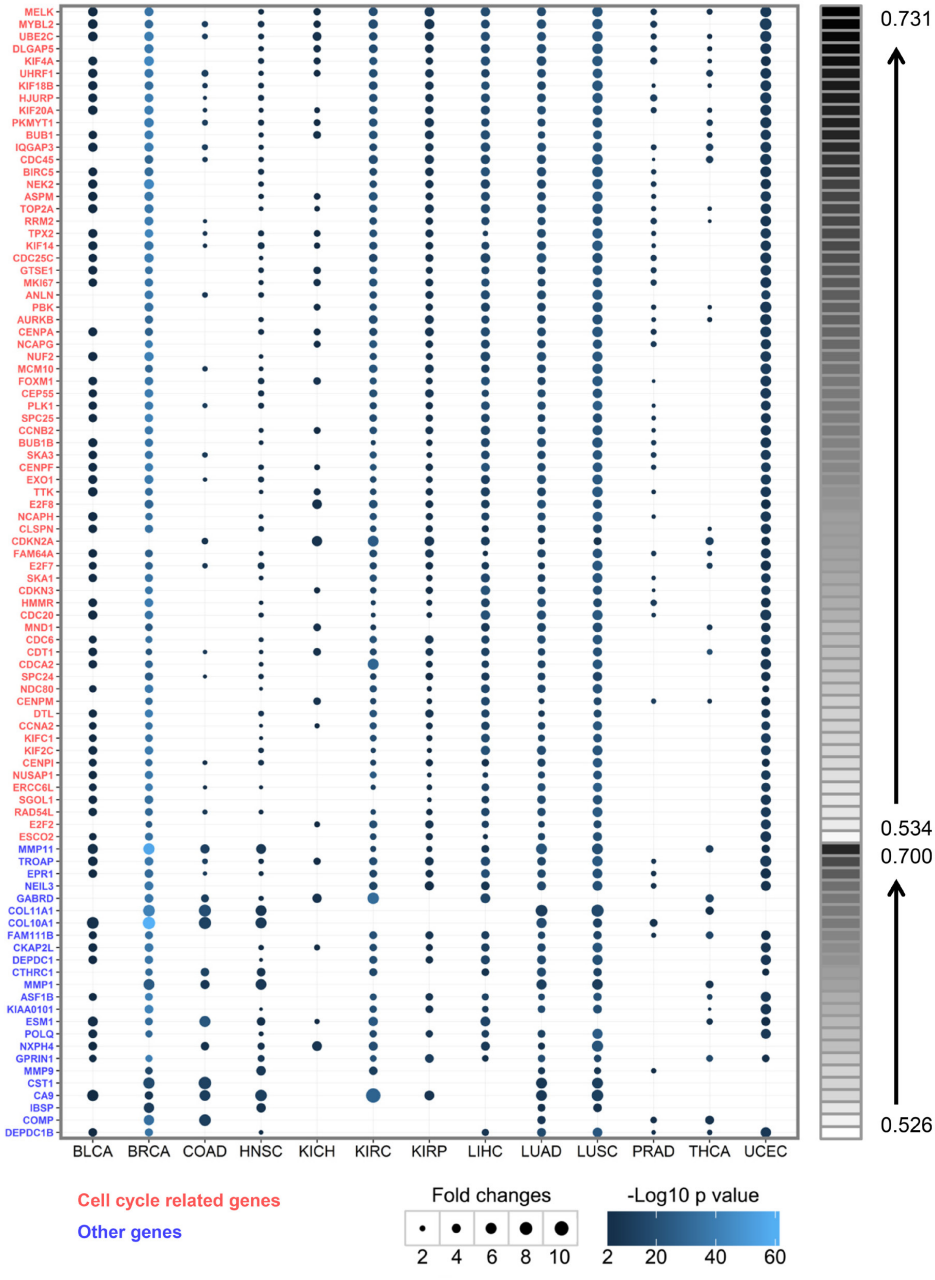
Pan-cancer genes in 13 cancer types Expression levels of pan-cancer genes were compared between tumors and paired normal samples. The sizes of points represent log2(fold-change) and the colors of points represent negative log10-transformed *p* value. Only points with *p* value < 0.01 and log2(fold-change) ≥ 1.5 are drawn. The red color on the left shows cell cycle-related genes while the blue color shows other genes. The right bar shows the proportions of samples with log2(fold-change) ≥ 2 in all 633 samples for each gene. The 92 genes are ordered according to the proportions.

In our consensus clustering result, a small number of genes were consistently upregulated in tumor tissues (Figure 1). We scanned all genes by calculating proportions of samples with gene log2(fold-change) ≥ 2 in all 633 samples and 92 genes ranking in the top 1% were retained (Figure 4). The differential expression significance was also calculated by Student's *t* statistics. All 92 genes were upregulated with log2(fold-change) ≥ 2 in more than half tumor samples across multiple cancer types. The top one gene, MELK was upregulated in 73.1% samples and across 12 cancer types. Interestingly, MELK has been reported as a novel oncogenic kinase and a promising selective therapeutic target for basal-like breast cancer recently [40]. Our result suggests that MELK may be a pan-cancer oncogenic kinase and a promising selective therapeutic target for multiple cancer types including uterine corpus endometrial carcinoma, bladder urothelial carcinoma, lung cancer, liver cancer, and kidney cancers. Using Gene Ontology Consortium [41], we found that more than two thirds genes (68/92) were included in cell cycle-related biological processes. The remaining 24 genes were involved in other biological processes (Figure 4).

Among the 92 genes, 10 genes including BUB1B, CCNB2, CDC25C, CDKN2A, COL11A1, FAM111B, MKI67, NDC80, NEK2 and TTK have previously been identified to be cancer driver genes *via* NCG 5.0 [42] (Supplementary Table 2). Thirty-three genes have been reported to promote proliferation or invasion in cancer, 48 genes are potential prognostic biomarkers, and 4 genes are related to drug resistance (Supplementary Table 2). The rest 25 genes including CENPI, COMP and TROAP may be novel cancer-associated genes that are worthy of validation in further studies.

### In-silico analysis and experimental validation of two pan-cancer genes

Since most pan-cancer genes have no explicit functions in tumor development, we validated the functions of two pan-cancer genes, FAM64A and TROAP, in tumor progression. FAM64A and TROAP were selected based on the following five criteria: (1) they were rarely reported in previous researches; (2) they have been detected in tumor tissues; (3) their functions in cancer progression are still unclear; (4) they belong to cell cycle related genes and other genes, respectively; (5) their proportions of samples with log2(fold-change) ≥ 2 in all 633 samples ranked at the top in all the genes satisfying criteria (1-4). Among cell cycle related genes, FAM64A was reported only in five papers and one paper reported its association with poor prognosis of triple-negative breast cancer [43]. Among other genes, TROAP was reported only in four papers and one paper reported its detection in serous ovarian adenocarcinoma [44]. FAM64A and TROAP met the criteria (1-5) and therefore were selected for further experiments.

**Figure 5:**
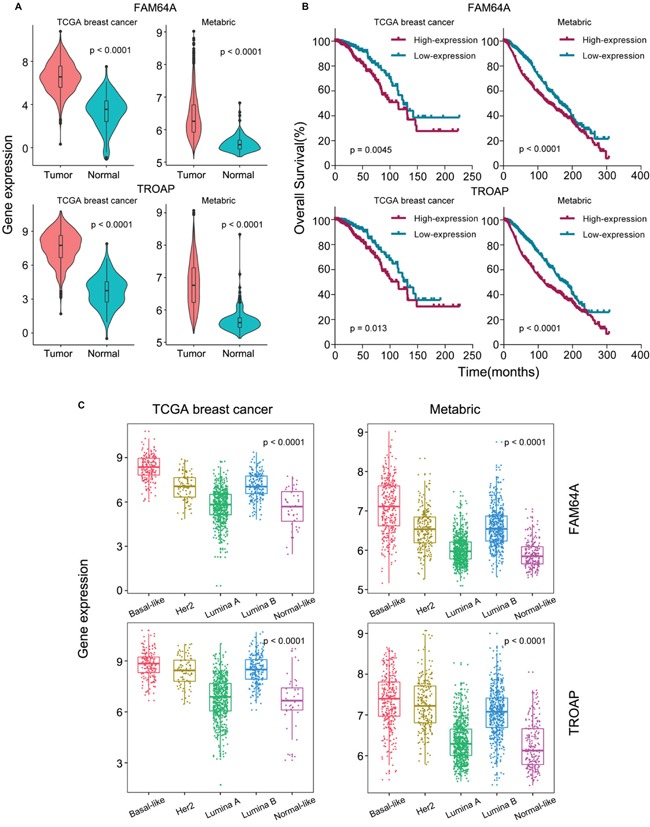
Analysis of pan-cancer genes FAM64A and TROAP **(A)** The expression levels of FAM64A and TROAP were compared between tumor and normal tissues in TCGA breast cancer and METABRIC datasets, respectively. The Student's *t* statistic was used to evaluate statistical difference. **(B)** Kaplan-Meier curves of FAM64A and TROAP in TCGA breast cancer and METABRIC datasets. Samples were stratified according to gene expression levels. The cutoff values were derived from the Cutoff Finder. **(C)** The expression levels of FAM64A and TROAP in PAM50 subtypes in TCGA breast cancer cohort and METABRIC dataset. One-way ANOVA was performed to evaluate the statistical difference among the five PAM50 subtypes.

We first analyzed gene expression levels between tumor and normal tissues of these two genes in TCGA breast cancer and METABRIC dataset [45]. The results showed that FAM64A and TROAP were significantly upregulated in tumor tissues (*p* value < 0.0001, Figure 5A). We then performed survival analysis and found that high expression of FAM64A and TROAP were significantly correlated with poor survival (FAM64A in TCGA *p* value = 0.005 and in METABRIC *p* value < 0.001, TROAP in TCGA *p* value = 0.013 and in METABRIC *p* value < 0.001, Figure 5B). We also found that FAM64A and TROAP could predict overall survival in multiple other cancer types (Supplementary Figure 7A, 7B). We further performed one-way ANOVA to test the significance of group differences among the PAM50 subtypes of breast cancer. We found that both FAM64A and TROAP were differentially expressed among five subtypes. Moreover, FAM64A and TROAP were most highly expressed in basal-like subtype (Figure 5C). The in-silico analysis results suggest that FAM64A and TROAP may promote the development of breast cancer, especially the basal-like subtype.

**Figure 6:**
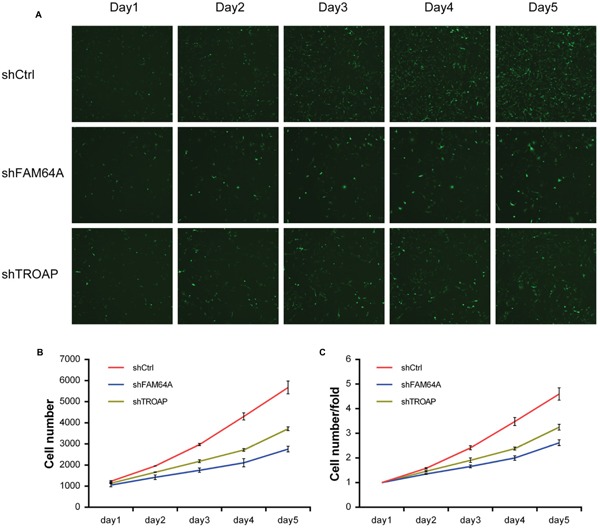
Validation of proliferation function of FAM64A and TROAP in MDA-MB-231 cell line **(A)** FAM64A and TROAP were knocked down by transfecting lentivirus expressing both shRNA and green fluorescent protein. Cell cytometry was performed every day for five days using the Celigo system. **(B)** The proliferation curves showed the average and standard deviation of cell numbers in three wells for FAM64A knockdown group, TROAP knockdown group and control group for five days. **(C)** The cell number fold was calculated by dividing cell number of a given day by the previous day. The cell number fold of the first day was set to 1. The *p* values of paired one-tail t-test are 0.03798 and 0.04098 for FAM64A and TROAP, respectively.

We conducted RNAi experiments to validate the effect of *FAM64A and TROAP* in breast cancer cell proliferation. Short hairpin RNAs (shRNA) were designed to knock down the expression levels of FAM64A and TROAP in MDA-MB-231 cells. RT-PCR experiments indicated that shRNA interference reduced FAM64A and TROAP expression levels by 80.7% and 59.4% (Supplementary Figure 8). Then we measured cell growth of the knockdown groups and the control group for five days. Compared with the control group, the groups with knockdown of FAM64A and TROAP exhibited significantly slower proliferation (1.76- and 1.41-fold, respectively) (Figure 6). These results suggest that inhibiting either FAM64A or TROAP can suppress the growth of breast cancer cells.

## DISCUSSION

In this study, we performed a pan-cancer analysis based on transcriptional response profiles of 633 paired tumor-normal samples from 13 cancer types. Two interesting results were obtained. On one hand, we identified 10 clusters with different transcriptional response patterns and pathways. HNSC and BRCA were both separated into two distinct subtypes. All the BLCA, UCEC, basal-like breast cancer, and one HNSC subtype were grouped together and formed a mixed cluster. On the other hand, we also identified 92 pan-cancer genes that were upregulated across multiple cancer types. Knockdown of two of these pan-cancer genes inhibited the growth of breast cancer cells. Our transcriptional response-based pan-cancer analysis provides novel perspectives of cancer molecular mechanisms.

Tumor is a complex genomic disease that develops due to accumulating mutations. Adjacent normal tissues are good controls since they contain genomic, transcriptomic and other omics information of the same individual. Transcriptional responses represent the changes of gene expression levels between tumor and adjacent normal tissues, which can reduce both individual differences and the impact of tissue-specific genes. A recent study of association between paired normal samples and patient survival revealed that paired normal tissues offered additional information on patient prognosis [46]. Our transcriptional response-based pan-cancer analysis obtained several novel and interesting results, which may deepen the understanding of tumorigenesis and cancer progression.

In our analysis, HNSC was classified into two distinct subtypes. HNSC Subtype 1 was enriched in cell cycle-related pathways, which had a good prognosis; HNSC Subtype 2 was enriched in cell adhesion-related pathways, and had a poor prognosis. However, all HNSC samples clustered together in tumor-only pan-cancer analysis. This result suggests that the transcriptional responses between tumor and adjacent normal tissues can highlight the differences of gene expression and pathway activity in each subtype, whereas tumor-only expression misses the information. A study concentrated on oral carcinoma by Suzanne *et al*. also found that overexpression of a 4-gene signature (MMP1, COL4A1, P4HA2, and THBS2) in histologically normal surgical margins could identify patients at high risk of recurrence [20]. Notably, HNSC subtype 1 might be suitable for chemotherapy, since tumors that overexpress cell cycle genes are sensitive to chemotherapy. Our analysis suggests that HNSC subtypes could be characterized with paired tumor-normal tissue samples and may be associated with therapeutic regimens.

During the pan-cancer analysis, we also identified 92 pan-cancer genes which were upregulated across multiple cancer types. Some of these pan-cancer genes have previously been demonstrated to be driver genes, oncogenes, or even candidate therapeutic targets, such as MELK [40]. Most of these genes are only reported to be potential biomarkers, whose functions in cancers are still elusive. To validate their functions, we knocked down the expression levels of FAM64A and TROAP in basal-like breast cancer cells and found that the growth of cancer cells was significantly inhibited. Our results indicate that the pan-cancer genes without known functions in cancer may promote tumor development and are worthy of further validations.

The main limitation of our analysis is the small size of paired tumor-normal samples for each cancer types. Therefore, the pan-cancer clusters obtained in this study should be validated in a larger sample size with paired tumor and adjacent normal tissues. Nonetheless, our analysis highlights the importance of normal samples in pan-cancer research and provides novel perspectives for cancer research.

## MATERIALS AND METHODS

### Data preparation

All samples in this study were obtained from the Cancer Genome Atlas (TCGA) project. Transcriptomic data from different cancer types were downloaded from the Broad Institute GDAC FireBrowse (TCGA data version 20141017, http://firebrowse.org/). All gene expression data were generated from Illumina HiSeq platform and quantified using RNA-Seq by Expectation Maximization (RSEM) [47]. Samples with both tumor and paired normal tissues were selected, and cancers with less than 15 tumor-normal paired samples were discarded. Finally, 633 samples from 13 cancer types (BLCA, BRCA, COAD, HNSC, KICH, KIRC, KIRP, LIHC, LUAD, LUSC, PRAD, THCA, and UCEC) were included in this study. Clinical information data were downloaded from the Broad Institute GDAC FireBrowse (http://firebrowse.org/). *METABRIC* data were downloaded from European Genome-phenome Archive (Study Accession: EGAS00000000083 https://www.ebi.ac.uk/ega/).

### Gene expression profile processing for tumor-normal paired analysis

Gene expression profiles of RSEM data from both tumor and normal tissues were normalized within-sample to a fixed upper quartile. Next, upper quartile-normalized data were log2-transformed. This processing procedure made the integrated analysis of gene expression profiles feasible and has been widely taken in the previous pan-cancer analysis [11, 13]. Then we plotted the empirical cumulative distribution of log2-transformed gene expression values in tumor and normal tissues, respectively (Supplementary Figure 9). We found that < 5% of the gene expression values were < 0 and < 5% of the gene expression values were > 12 in both tumor and normal tissues. Therefore we truncated the log2-transformed gene expression values < 0 to 0 and > 12 to 12 in order to avoid extremely small or large transcriptional responses which will affect subsequent analysis. Finally, transcriptional responses for the tumor-normal paired analysis were represented by the log2(fold-changes) between tumor and matched normal data.

### Consensus clustering

To generate a persistent clustering result, ConsensusClusterPlus R-package [48] was used to identify clusters using 1,000 iterations (*reps*), 80% sample resampling (*pItem*) from 2 to 20 clusters (*k*) using hierarchical clustering algorithm (*clusterAlg*). The distance matrix was set to Pearson correlation (*distance*) and linkage function was set as wald. D (innerLinkage) and average (finalLinkag). In order to select optimal cluster number *k*, we calculated the empirical cumulative distribution (CDF) and the proportional area change under CDF (Δ(*k*)). According to the Δ(*k*) vs *k* plot, the *k* where Δ(*k*) started to approach zero was optimal. We also plotted the heatmap of consensus matrix at *k* to observe whether boundaries of each cluster were sharp. Considering the results of the Δ(*k*) *vs k* plot and the heatmap, we determined the optimal cluster numbers.

### Clustering procedure for tumor-only analysis

In the tumor-only analysis, only 633 tumor samples were used. Gene expression data were derived from the upper quartile-normalized RSEM data of tumor tissues. The top 4,000 most variable genes were selected according to median absolute deviation.

The number 4,000 was determined according to previous pan-cancer works and our experience. In previous pan-cancer works, top 1,500 [11] and top 6,000 [13] most variable genes were selected for clustering, which both resulted in valuable findings. We performed consensus clustering on tumor-only samples with gene numbers varying from 1,500 to 6,000, with 500 as a step. We investigated the consistency of the clustering results pair wisely between different gene numbers. All the Rand indexes were very high, ranging from 0.994 to 1.000 (Supplementary Figure 3B). This result suggests that different gene numbers have little impact on the tumor-only clustering result. We therefore chose to use the median gene number, 4,000 in the tumor-only analysis.

### Gene set enrichment analysis

Gene set enrichment analysis was performed using GSEA tool. Canonical pathways were downloaded from Molecular Signatures Database (MsigDB version 5.1) [31]. Transcriptional response profiles of 633 samples were input into GSEA and gene sets enriched in each cluster were identified by comparing one to all the rest clusters. Finally, gene sets with nominal *p* value < 0.01 were selected and shown in Figure 3C. Differentially expressed genes between two HNSC subtypes were identified *via* limma R-package [49]. Genes in either subtypes with *p* value < 0.01 and log2(fold-change) ≥ 1.5 were retained. DAVID functional annotation tool was used to annotate the genes. GO terms with Bonferroni-corrected *p* value < 0.01 were considered as the dominant pathways.

### Cell culture and transfection

Human breast cancer cell line MDA-MB-231 (ATCC, Manassas, VA) was cultured in 6-cm culture dishes in DMEM medium (Corning; NY, USA) (4ml per dish) with fetal bovine serum (FBS; Ausbian, Australia). Cells were incubated at 37°C in a humidified 5% CO_2_ atmosphere. All cells were used during the exponential phase of growth.

Confluent MDA-MB-231 cells were seeded at a density of 1,500-2,500 cells/well in a 96-well plate. When 20-30% confluence was reached, cells were transfected with shRNA lentivirus (GeneChem, Shanghai, China) containing green fluorescent protein (GFP) at 10 moi. In order to guarantee the efficiency of gene knockdown, we designed three shRNA sequences per gene targeting different sites and mixed them together at similar ratios. The target sequences of the shRNA knockdown constructs for FAM64A were 5’-GTCCCAAGAGCTAGATGAA-3’, 5’-CACCCATTACGGCGATCAA-3’, and 5’-TGCCAA AGTGGCACCAAGT-3’. The target sequences of the shRNA knockdown constructs for TROAP were 5’-AACCAAGATCCAAGGAGAT-3’, 5’-CGCCG TGGACCAGGAGAACCA-3’, and 5’-AAGGAGATG GGTGCAGAAACC-3’. The sequence of the control vector was 5’-TTCTCCGAACGTGTCACGT-3’. Cells were incubated for 24 hours, and the media was changed to remove remaining transfection reagent. About 2-3 days later, after fluorescence intensity had increased to 70-90%, cells were cultured further to reach 70-90% confluency and collected for cell cytometry analysis. The estimated transfection efficiency was 70-90%.

### Cell cytometry and image analysis

Stained MDA-MB-231 cells were plated in 96-well plate at a density of 1,000 cells/well. To ensure reproducibility, shFAM64A and shTROAP transfected cells and control cells were plated in 3 wells, respectively. Then the plates were incubated for 24 h under 5% CO_2_ at 37 °C. After that, the plates were fixed and imaged with the adherent cell cytometry system Celigo acquiring four images per well for 5 days. Images were acquired for each fluorescence channel, using suitable filters and 20 × objective. Through optimizing the analysis setting parameters, the accurate number of cells in each field was counted accurately. Cell numbers of each well were represented by the accumulation of four fields. The average and standard deviation of cell numbers of the three wells for FAM64A and TROAP knockdown groups and control group were calculated during five days.

### Statistical analysis

All statistical analyses were performed using R programming platform. The R package limma was employed for differential expression analysis. The R package survival was used for survival analysis. Kaplan-Meier curves and log-rank test were used to assess differences between survival distributions. We classified patients into two groups based on gene expression according to the Cutoff Finder application [50]. The Student's t test was used to evaluate the statistical significance of differences between tumor and normal expression data in TCGA breast cancer and METABRIC cohort. One-way ANOVA was used to compare the differences among PAM50 subtypes. Kaplan-Meier curves were plotted with GraphPad Prism 6. Other plots were generated using R packages ggplot2 and pheatmap.

## SUPPLEMENTARY MATERIALS FIGURES AND TABLES




