# Supplementary material for: Transcriptional response profiles of paired tumor-normal samples offer novel perspectives in pan-cancer analysis
Source: Oncotarget. 2017 Apr 20;8(25):41334–47. doi: 10.18632/oncotarget.17295 (PMC5522216; doi:10.18632/oncotarget.17295)
Supplement: Supplementary file 1 [file oncotarget-08-41334-s001.pdf]

Transcriptional response profiles of paired tumor-normal samples offer novel perspectives in pan-cancer analysis

SUPPLEMENTARY MATERIALS

SUPPLEMENTARY TABLES AND FIGURES

Supplementary Table 1: Summary of the 13 cancer datasets

Cancer Type	Abbreviation	Number
Bladder Urothelial Carcinoma	BLCA	19
Breast Cancer	BRCA	111
Colon Adenocarcinoma	COAD	41
Head and Neck Squamous Cell Carcinoma	HNSC	41
Kidney Chromophobe Renal Cell Carcinoma	KICH	25
Kidney Clear Cell Renal Cell Carcinoma	KIRC	72
Kidney Renal Papillary Cell Carcinoma	KIRP	32
Liver Hepatocellular Carcinoma	LIHC	50
Lung Adenocarcinoma	LUAD	57
Lung Squamous Cell Carcinoma	LUSC	51
Prostate Adenocarcinoma	PRAD	52
Thyroid Carcinoma	THCA	59
Uterine Corpus Endometrial Carcinoma	UCEC	23
Total		633

Supplementary Table 2: Pan-cancer functional genes

See Supplementary File 1

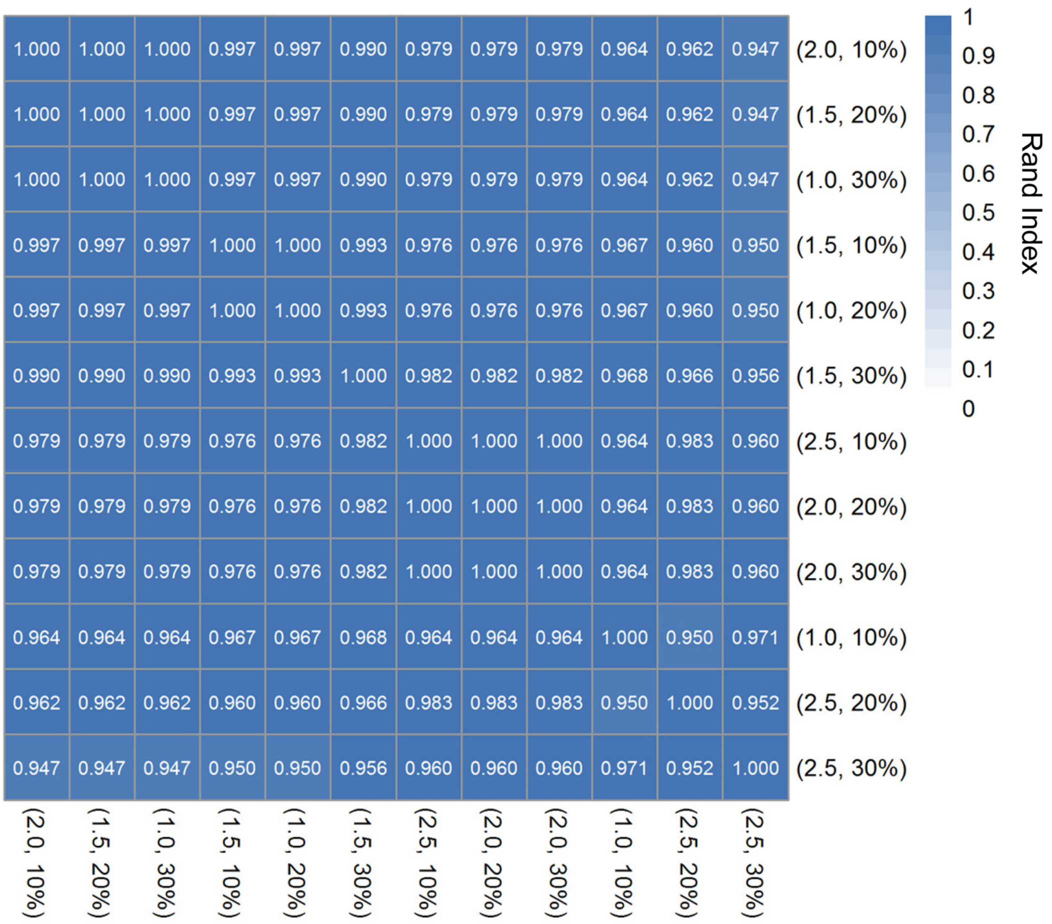

Supplementary Figure 1: Pairwise Rand indexes between each combination of two cutoff values. The former cutoff value in brackets corresponses to log2(fold-change). The latter cutoff value corresponses to sample percent. Rows and columns were sorted in decreasing order of the Rand indexes of clustering results between each combination and (2.0, 10%). All the Rand indexes are very high, ranging from 0.947 to 1.000. This result suggests that clustering patterns derived from different combination of two cutoff values are consistent.

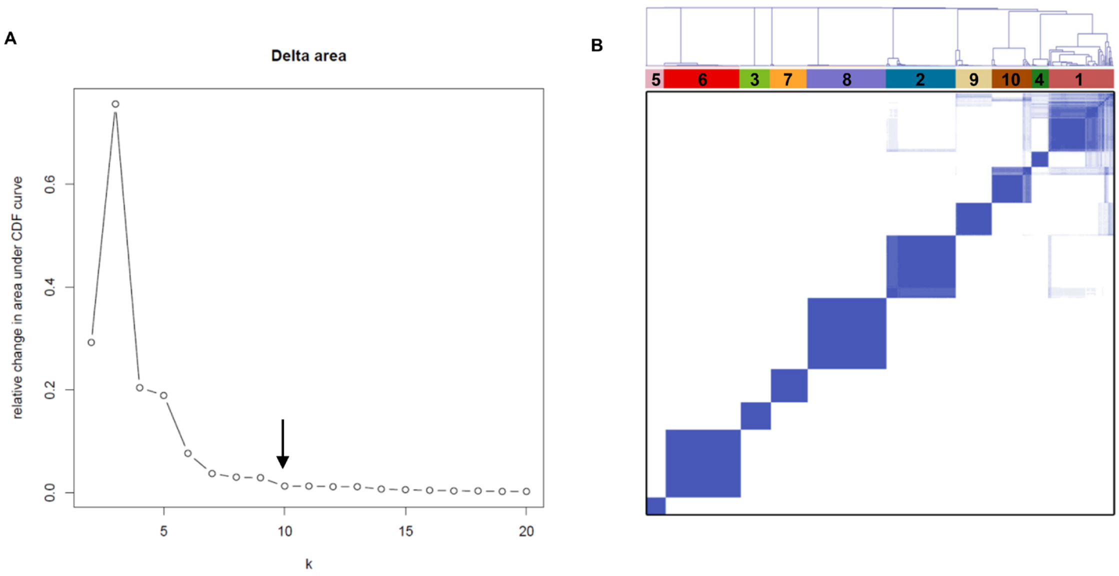

Supplementary Figure 2: Selection of the optimal cluster number. **(A)** The Δ(k) vs k plot. **(B)** Heatmap shows the consensus matrix at $k = 10$.

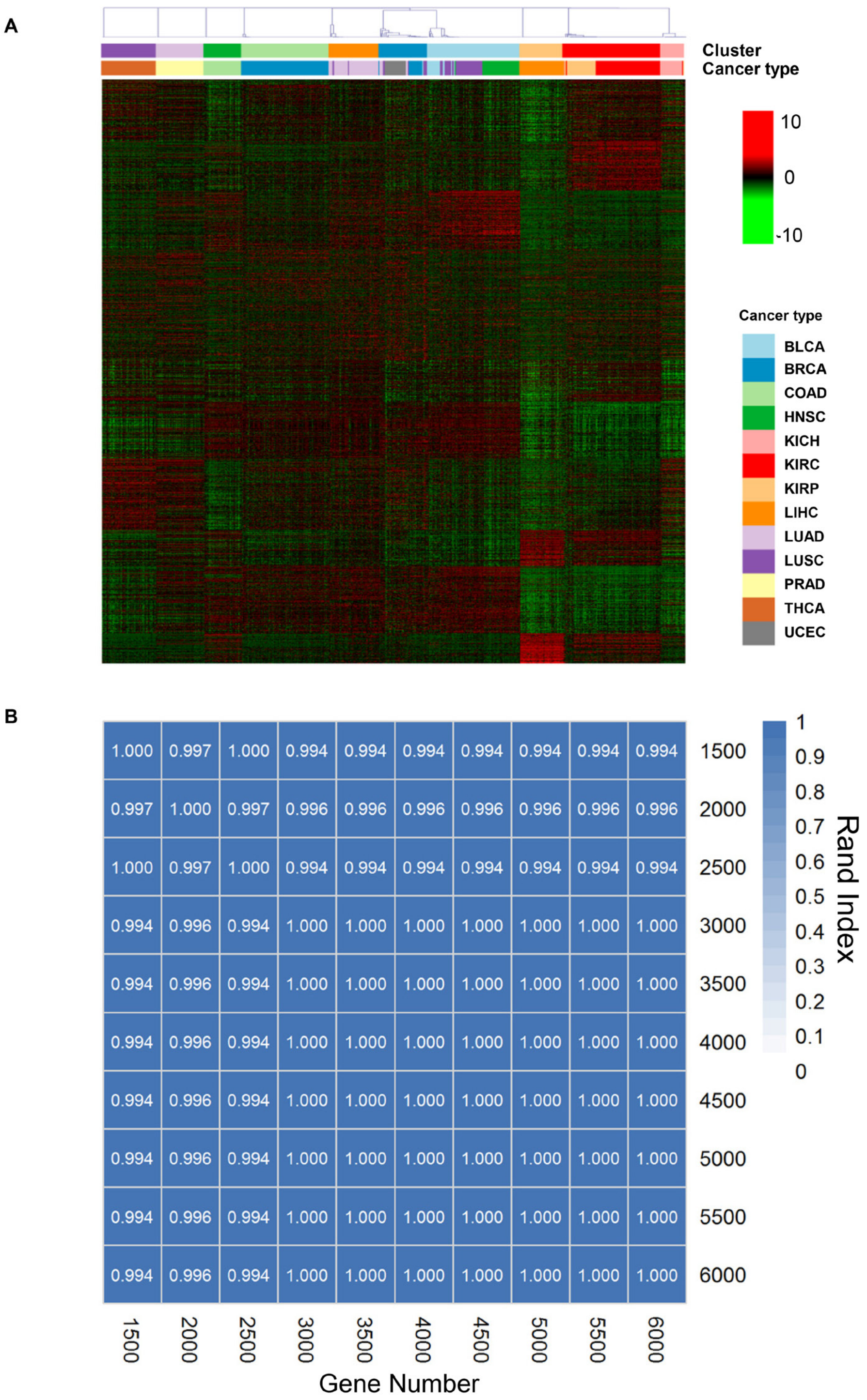

Supplementary Figure 3: Consensus clustering based on tumor-only expression profile. **(A)** Heatmap shows the pattern of tumor-only gene expression profile derived from consensus clustering algorithm. Rows indicate genes and columns indicate samples. The 10 clusters identified are shown by different colors in the top bar. Cancer types are shown by different colors in the second bar. **(B)** Pairwise Rand indexes between different gene numbers.

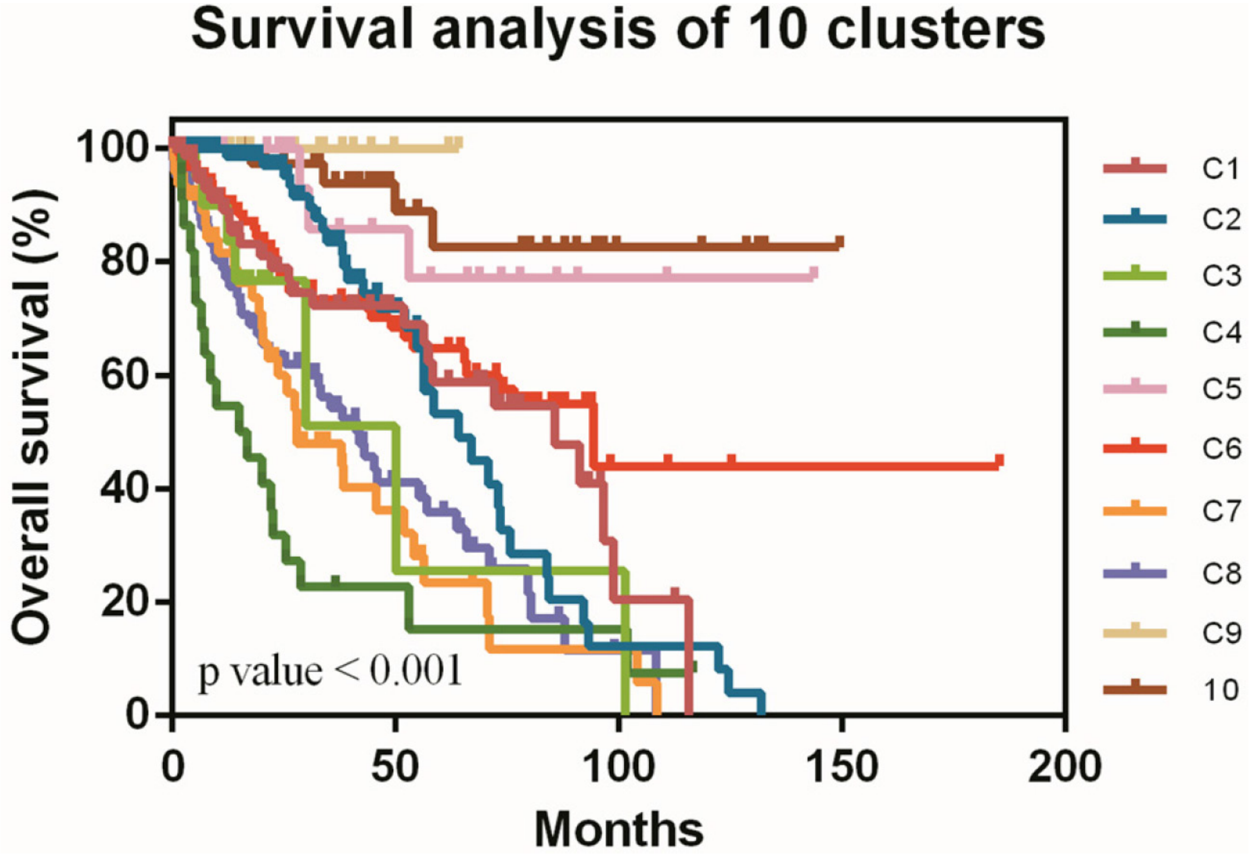

Supplementary Figure 4: Kaplan-Meier survival analysis of 10 clusters.

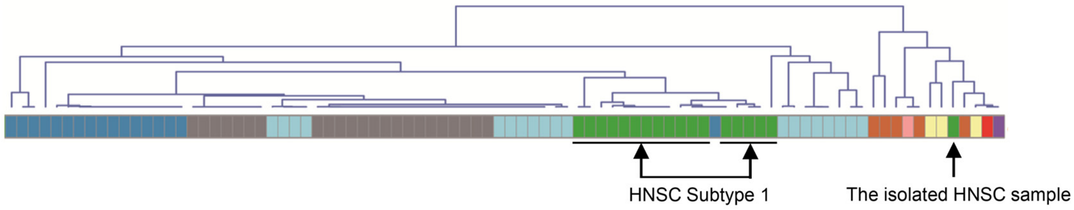

Supplementary Figure 5: Hierarchal tree of C1. The isolated HNSC sample is far away from HNSC Subtype 1.

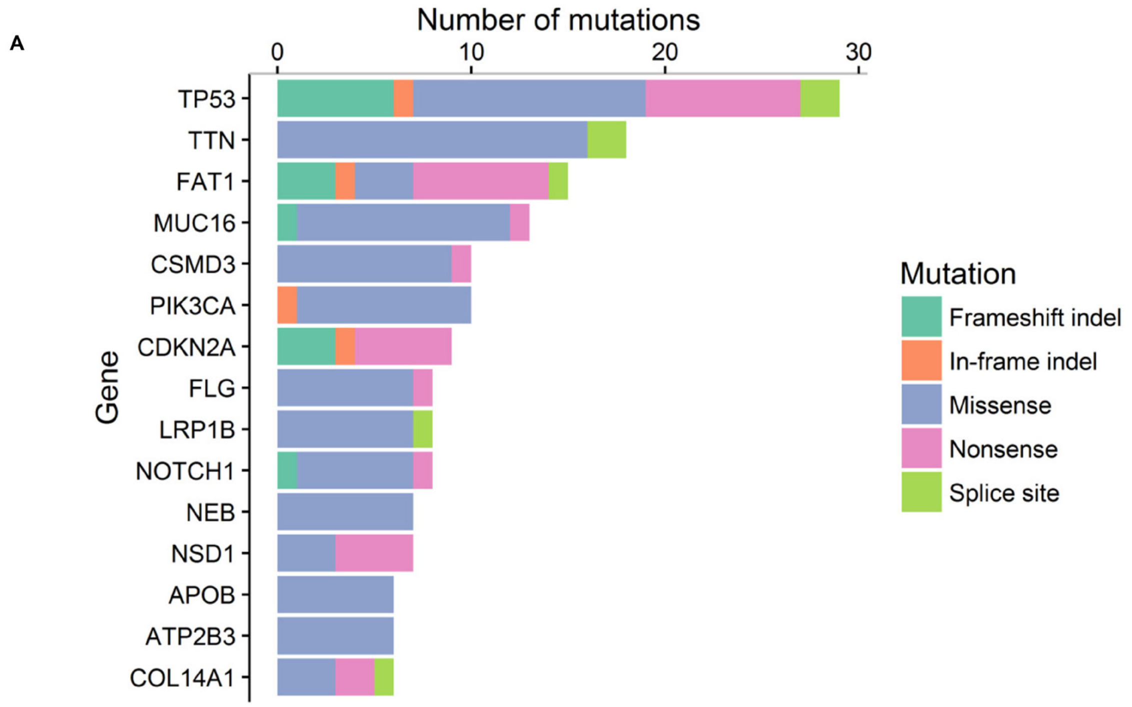

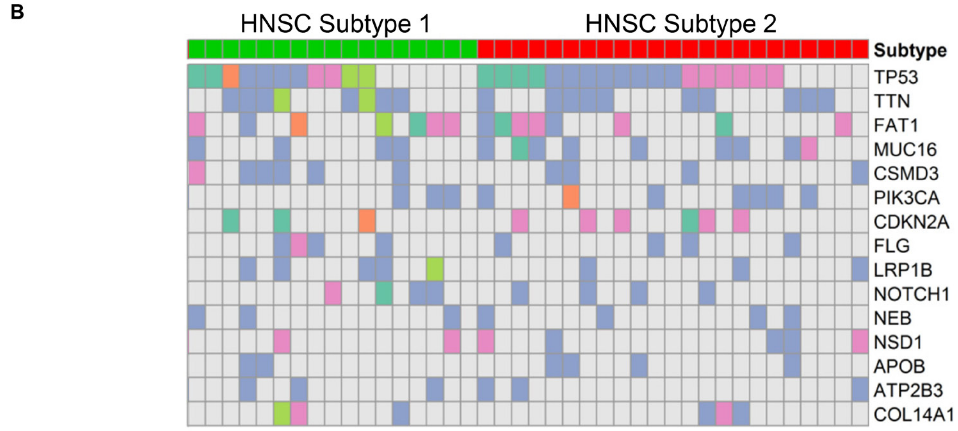

Supplementary Figure 6: Gene mutations in HNSC. (A) Numbers of mutations for the top 15 genes. Types of mutations are shown in the right bar. **(B)** Mutation types of the top 15 genes for each sample in HNSC Subtype1 and Subtype 2.

A

FAM64A

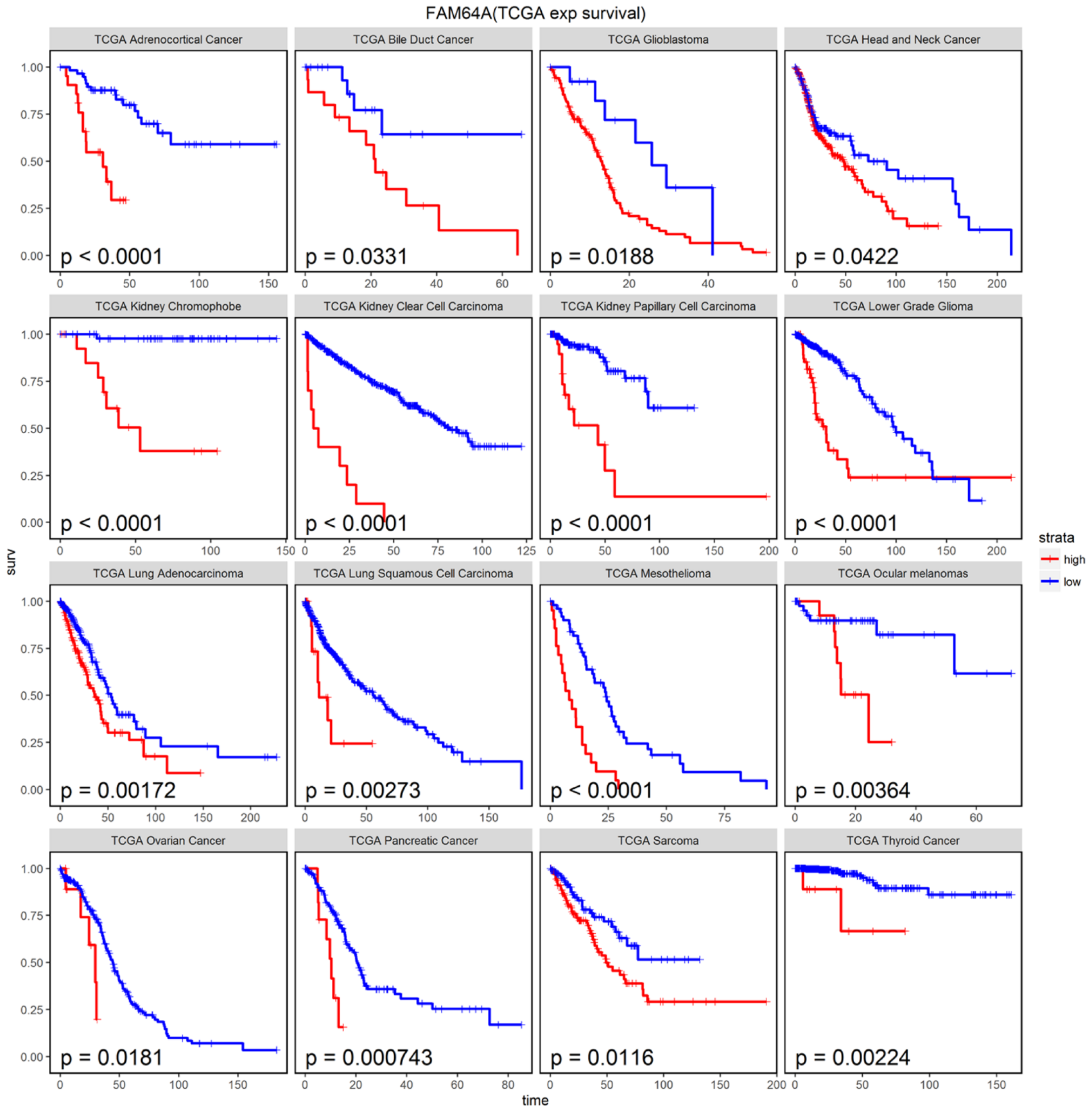

(*Continued*)

B

TROAP

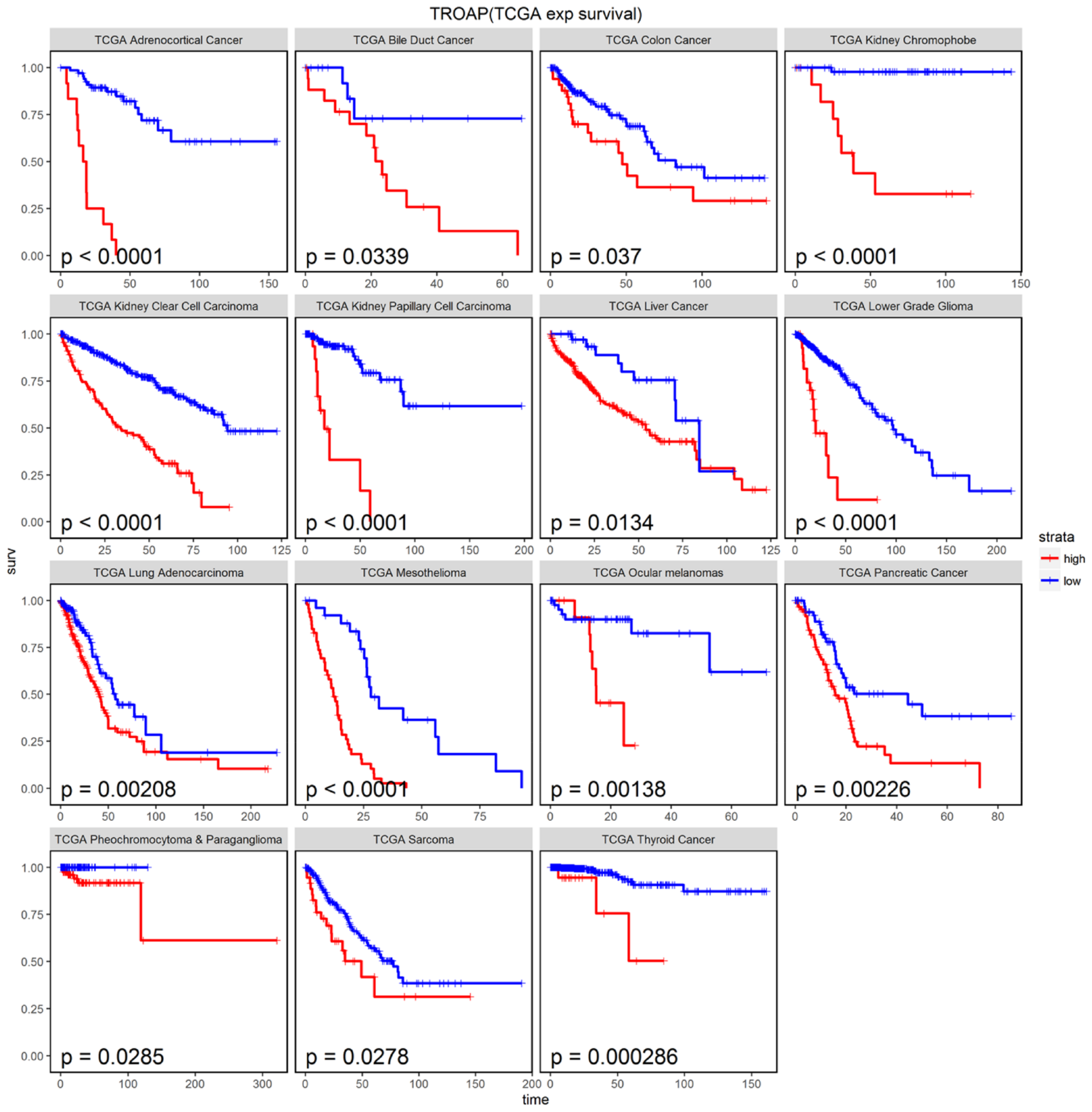

Supplementary Figure 7: The relationship of FAM64A and TROAP with overall survival in multiple cancer types. (A) and **(B)** shows Kaplan-Meier survival curves of FAM64A and TROAP in multiple cancer types. Only analysis results with *p* value < 0.05 are shown.

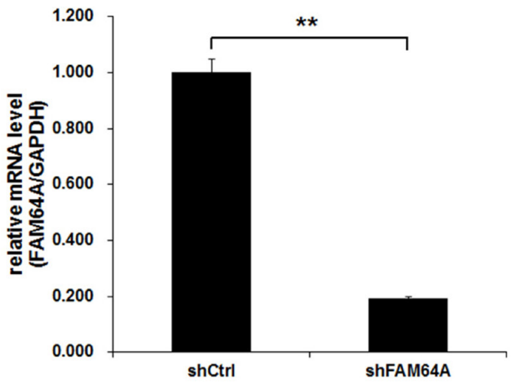

Supplementary Figure 8: Efficiency of FAM64A-shRNA and TROAP-shRNA interference measured by RT-PCR. FAM64A and TROAP gene expression levels were reduced by 80.7% and 59.4% with shRNA interference. $**p$ value < 0.05.

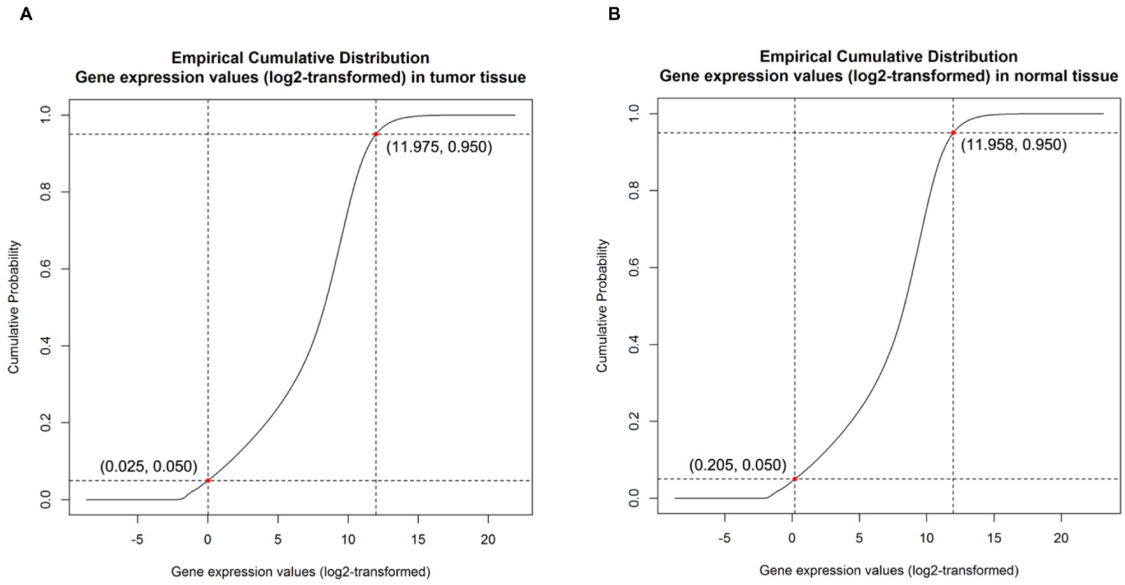

Supplementary Figure 9: Empirical cumulative distribution of log2-transformed gene expression values. (A) Empirical cumulative distribution of log2-transformed gene expression values in tumor tissues. **(B)** Empirical cumulative distribution of log2-transformed gene expression values in normal tissues. The horizontal dotted lines in **(A)** and **(B)** are drawn at 5% and 95% of y axis. The red points are intersections of the horizontal dotted lines and the empirical cumulative distribution curves. The vertical dotted lines in **(A)** and **(B)** are drawn across the intersections.
